# Augmented reality system for endoscopic pituitary surgery with automatic registration

**DOI:** 10.1007/s11548-025-03384-3

**Published:** 2025-05-11

**Authors:** Aure Enkaoua, João Ramalhinho, Mobarakol Islam, Hani J. Marcus, Matthew J. Clarkson

**Affiliations:** 1 UCL Hawkes Institute, Department of Medical Physics and Biomedical Engineering, University College London, London, UK; 2 National Hospital for Neurology and Neurosurgery, London, UK

**Keywords:** Augmented reality, Transsphenoidal pituitary surgery, Minimally invasive surgery, Endoscopic surgery

## Abstract

**Purpose:**

Endoscopic pituitary surgery is a minimally invasive technique to remove pituitary tumours through the nose. Currently, image guidance may be used in the form of a tracked pointer to help surgeons navigate the region and avoid damage to critical structures. However, the pointer method is mentally demanding as the pointer location is displayed in a different modality and disrupts the surgical workflow due to the setup time and frequent tool removal.

**Methods:**

We propose an Augmented Reality (AR) system where information from the pre-operative scan is displayed directly onto the endoscopic video. Our system features an on-board tracking system, allowing for the registration process to be performed automatically.

**Results:**

We evaluated the accuracy of our system and compared it to an AR system that uses an infrared (IR) camera to track an endoscope with reflective markers. Our system gave an accuracy of 1.1 (± 0.4) mm, compared to 2.4 (± 0.9) mm in the IR-tracked endoscope approach.

**Conclusion:**

Our Augmented Reality system is a more compact and transportable setup which outperformed the IR-tracked endoscope. The automatic registration method can save time in the operating room as well as increase AR overlay accuracy, improving the translation of these technologies.

## Introduction

Endoscopic pituitary surgery is a minimally invasive technique performed to remove pituitary tumours by accessing the base of the skull through the sphenoid sinus with the aid of an endoscope [1]. While it offers advantages over open skull-base surgery [2], it remains high risk due to the close proximity of the pituitary gland to the dense surrounding anatomy.

In order to understand the orientation of the anatomy and avoid damage to critical structures, surgeons currently use a tracked pointer, whose tip location can be synchronously mapped on a pre-operative MRI scan [3]. However, as the pointer location is shown on a 3D MRI scan but the surgery is seen on a 2D endoscopic video, this method increases cognitive load as the surgeons have to mentally map between different modalities and dimensions. Additionally, the limited nasal cavity space requires the tool to be removed every time a localisation is required, disrupting workflow and delaying the surgery.

Augmented Reality (AR) has been explored as a solution, overlaying pre-operative data on live endoscopic video, with approaches using IR-reflective [4] or visual [5] markers. However, current methods either depend on markers being visible in the pre-operative scan [6, 7], require intra-operative scans to align the patient’s anatomy with the navigation system [8], or lack the required precision for pituitary surgery [5] [3]. Fully trackerless systems [9, 10] enable a less complex setup by removing the reliance on external markers. However, they face challenges such as featureless anatomy, limited training data, and hardware constraints, as these techniques would require powerful computational resources in order to work in real time.

## Methods

We present the first system in endonasal surgery to integrate an on-board tracking system. We compare our system to an IR-tracked endoscope approach, evaluating the overlay accuracy using predefined markers placed in a phantom model.

The phantom used for evaluating both systems was the UpSurgeOn BrainBox TNS model,^1^ used for training and simulating an endoscopic pituitary surgery. A CT scan of the phantom was obtained with the Medtronic O-arm CT O2 Intraoperative Imaging System, and its surface segmented and smoothed using 3D Slicer.^2^ A 1 cm grid of spherical markers was placed inside the phantom instead of the replaceable cavity, and the centres of these targets were identified on the CT scan (see Fig. 2). Target models were generated using these locations for the AR overlay. The phantom was held stationary relative to the markers with a custom-made structure simulating a Mayfield clamp. Once set up, both systems followed three key steps: calibration, registration, and tracking.

**Fig. 1:**
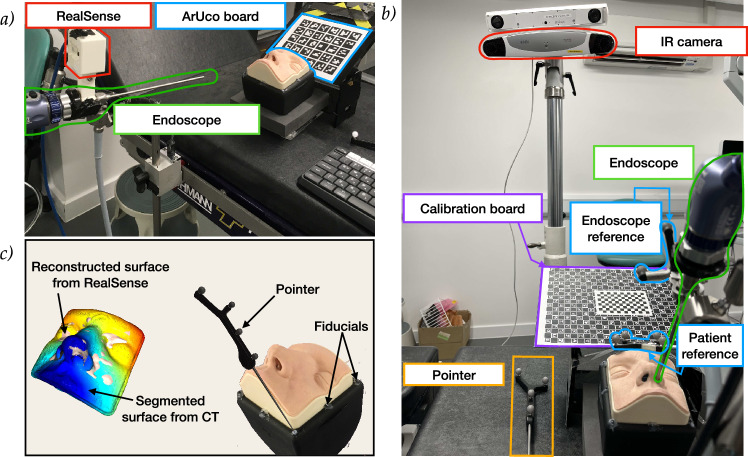
System overview of our system (**a**) and IR-tracked endoscope (**b**). The registration process of our system (left) and the IR-tracked (right) systems is depicted in (**c**). In our system (**a**), the ArUco board (blue) acts as the patient reference and is placed on the holder, where the calibration board is also placed. The board is tracked by the external RealSense camera (red), rigidly attached to the endoscope (green). The registration process of our system is shown on the left of (**c**). The RealSense camera is used to reconstruct the face of the phantom, which is registered to the CT scan. In the IR system (**b**), a patient reference (blue) is attached to the phantom holder, and a calibration board (purple) is used to calibrate the endoscope’s (green) intrinsic parameters and its pose relative to the endoscope reference (blue). The IR camera (red) tracks the pointer (orange) and reference markers. Registration as seen on the right of (**c**) is performed by placing the pointer at seven fiducials with known CT locations for point-based alignment

### Our system

Our proposed system can be seen in Fig. 1a. Our setup includes the INTEL® D405 RealSense™ stereo camera^3^ mounted on a standard Storz endoscope^4^ using a custom 3D printed bracket. An ArUco board was attached to the laser-cut holder that held the phantom stationary.

***Calibration:*** The intrinsic parameters of the endoscope were calibrated using a 5x9 ChArUco board with 20 mm squares, following Zhang’s method [11] as implemented in OpenCV [12]. The RealSense™ camera was calibrated using its built-in self-calibration method for the D400 series.^5^ Extrinsic calibration between the cameras was determined by recording frames where the board was visible from both the RealSense™ and endoscope cameras and calculating the relative transform using the two estimated poses.

***Registration:*** As seen on the left of Fig. 1c, to obtain the registration, the phantom was reconstructed using the RealSense camera. Using prior knowledge on the pose of the RealSense camera relative to the face, the segmented CT surface was oriented in the same direction as the reconstructed face. A robust point-to-plane ICP algorithm using Tukey loss $(k=0.1)$ was applied [13] with a distance threshold of 1 mm to mitigate the influence of outliers.

***Tracking:*** Once registered, the 3D model overlay was updated based on the pose obtained from the ArUco board at each frame.

### IR-tracked endoscope

The IR-tracked endoscope approach can be seen in Fig. 1b. An NDI^6^ camera was used to track IR-reflective markers attached to the endoscope and phantom.

***Calibration:*** The intrinsic and extrinsic calibration processes were performed using the SmartLiver software as described in [14].

***Registration:*** Seven fiducial markers were placed along the base of the phantom as seen on the right of Fig. 1c, and their locations were localised on the CT scan. During the registration process, these same fiducials were sampled using the tracked pointer tip and could therefore be matched to the CT samples using the procrustes point-based registration algorithm [15].

***Tracking:*** Once registered, the 3D model overlay was updated based on the pose obtained from the patient reference and endoscope reference markers.

## Experiment and results

We calculated the projected target registration error (TRE) of our system by measuring the average Euclidean distance between the true target value locations and the AR overlay of the targets. This error includes the calibration, registration, and tracking errors. The experimental setup was designed to mimic clinical conditions, with both systems tested under similar constraints of the endoscope position, lighting, and zoom settings.

**Fig. 2:**
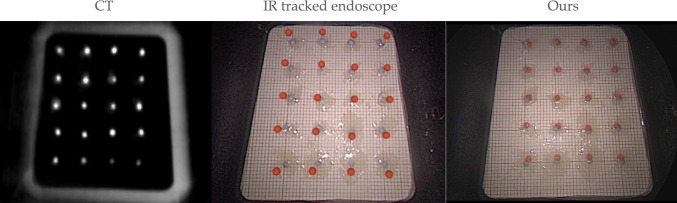
Accuracy comparison of IR-tracked AR (middle) and our system (right). Grey circles represent true target points, spaced 1 cm apart, while red dots show the AR overlay. The distance between the true target points (grey) and overlay points (red) represents the accuracy of either system. The CT scan with visible target points used for extracting red 3D locations is shown on the left

The accuracy of the systems was 2.4 (± 0.9) mm and 1.1 (± 0.4) mm for the tracked endoscope and our system, respectively. Figure 2 provides a clear visual representation of the accuracy evaluation, where the true target points are shown in grey and the AR overlay is shown by the red spheres.

## Discussion and conclusion

We have presented a new Augmented Reality system for performing pituitary surgery using an on-board tracking system. Our system is more affordable and compact than the current IR-tracked approach, making it more practical for widespread adoption by reducing both cost and the space required in the operating room.

Unlike the approach proposed by Onishi et al. [5], where cameras are placed in the operating room and may be obstructed by surgical staff, our system ensures an unobstructed view by mounting the tracking camera directly on the endoscope. Since the endoscope is inserted through the nose, the RealSense camera consistently faces the ArUco board placed on the patient’s head. Because the markers are attached to the patient, they cannot be blocked by surgeons moving around the room. Using an ArUco board instead of a single tag also allows the system to estimate the pose even when some markers are temporarily occluded.

In previous work, we showed that the IR tracking systems are not accurate enough for the scale of pituitary surgery [3]. Our proposed setup results in a higher accuracy since the tracking camera is placed nearer the reference. Furthermore, while both IR-tracked systems and the method by Onishi et al. [5] rely on two separate references for tracking, our system requires only a single reference, simplifying the setup.

A key advantage of our system is its automated registration pipeline. Automating this process can reduce setup time and minimise human error, particularly for inexperienced users.

### Limitations and future work

Our current evaluation is conducted on a stationary phantom, which allows for a controlled accuracy assessment. Although this study shows promise as a proof of concept, further evaluation is necessary to ensure reliability across different test models and real-world clinical settings.

The registration method used for our system suffers from limitations as it currently relies on a single reconstructed view. Even though this is enough for an initial evaluation with a static environment, it may be sensitive to cases where the reconstruction is poor. To improve robustness against such cases, we plan to explore combining multiple views to the reconstruction, which could fill missing areas in frames where there are occlusions, help detect outliers more effectively, and ultimately enhance the overall accuracy. Additionally, while our current registration method depends on prior knowledge of the expected orientation of the face, a more advanced approach will use real facial landmarks to estimate the initial orientation.

The registration technique used for the IR-tracked endoscope approach, as described in Sect. 2.2, relies on the fiducial markers being visible on the pre-operative scan. However, this method assumes the fiducials will remain fixed between the imaging session and the surgical procedure, which is not practical in a clinical setting. Instead, surface-based registration techniques, where points along the face of the patient are sampled for alignment, are typically used in surgery. Future work will compare the accuracy of our automatic registration and this more realistic registration method.

## Footnotes

1 UpSurgeOn: https://store.upsurgeon.com/

2 Slicer: https://www.slicer.org/

3 RealSense: www.intelrealsense.com/depth-camera-d405

4 Karl Storz: https://www.karlstorz.com/

5 RealSense self-calibration: https://dev.intelrealsense.com/docs/self-calibration-for-depth-cameras

6 NDI: https://www.ndigital.com/
